# Radiographic cup position following posterior and lateral approach to total hip arthroplasty. An explorative randomized controlled trial

**DOI:** 10.1371/journal.pone.0191401

**Published:** 2018-01-29

**Authors:** Christine Kruse, Signe Rosenlund, Leif Broeng, Søren Overgaard

**Affiliations:** 1 Department of Orthopaedic Surgery and Traumatology, Odense University Hospital and Institute of Clinical Research, University of Southern, Odense, Denmark; 2 Department of Orthopaedic Surgery and Traumatology, Zealand University Hospital, Køge, Denmark; Hvidovre Hospital, DENMARK

## Abstract

The two most common surgical approaches to total hip arthroplasty are the posterior approach and lateral approach. The surgical approach may influence cup positioning and restoration of the offset, which may affect the biomechanical properties of the hip joint.

The primary aim was to compare cup position between posterior approach and lateral approach. Secondary aims were to compare femoral offset, abductor moment arm and leg length discrepancy between the two approaches. Eighty patients with primary hip osteoarthritis were included in a randomized controlled trial and assigned to total hip arthroplasty using posterior approach or lateral approach. Postoperative radiographs from 38 patients in each group were included in this study for measurement of cup anteversion and inclination. Femoral offset, cup offset, total offset, abductor moment arm and leg length discrepancy were measured on preoperative and postoperative radiographs in 28 patients in each group. We found that mean anteversion was 5° larger in the posterior approach group (95% CI, -8.1 to -1.4; p = 0.006), while mean inclination was 5° less steep (95% CI, 2.7 to 7.2; p<0.001) compared with the lateral approach group. The posterior approach group had a larger mean femoral offset of 4.3mm (95% CI, -7.4 to -1.3, p = 0.006), mean total offset of 6.3mm (95% CI, -9.6 to -3; p<0.001) and mean abductor moment arm of 4.8mm (95% CI, -7.6 to -1.9; p = 0.001) compared with the lateral approach group. We found a larger cup anteversion but less steep cup inclination in the posterior approach group compared with the lateral approach group. Femoral offset and abductor moment arm were restored after total hip arthroplasty using lateral approach but significantly increased when using posterior approach.

## Introduction

One of the many factors that influence the outcome of total hip arthroplasty (THA) is component positioning. Improper cup positioning may be associated with dislocation [1–4], increased polyethylene wear [5, 6], reduced abductor muscle strength [7–10] and impingement [11]. The surgical approach may affect component positioning because the view of important anatomical landmarks differs [12]. The two most common surgical approaches for THA are the posterior approach (PA), which provides a good view of the proximal femur, and the lateral approach (LA) which offers a good view of the acetabulum [13].

The inferior view of the acetabulum during PA may influence the surgeons’ ability to place the cup precisely. PA has also been correlated with an increased risk of revision due to dislocation compared with LA [14–16]. Therefore, we found it relevant to investigate potential differences in cup positioning and offset changes between PA and LA as they might contribute to our understanding of any potential differences in clinical outcomes.

The aim of this explorative randomized study was primarily to compare cup anteversion and inclination between patients who had THA surgery performed with PA or LA. We hypothesized that anteversion would be greater and inclination would be smaller following PA because the surgeons aimed to reduce the risk of dislocation in patients treated with PA. The secondary aims were to compare preoperative to postoperative changes in femoral offset, cup offset, total offset, abductor moment arm and leg length discrepancy.

## Methods

### Study design

This study was based on radiographic data, which were collected as explorative outcomes during a randomized controlled trial (RCT). The RCT was designed as a prospective, blinded, parallel-group, superiority trial with balanced randomization (1:1). The main trial was registered at ClinicalTrial.gov (registration no.: NCT01616667) and a protocol has been published [17], additional the protocol for this study was published at www.protocols.io [18]. The study was reported according the CONSORT guidelines.

### Participants

**Fig 1:**
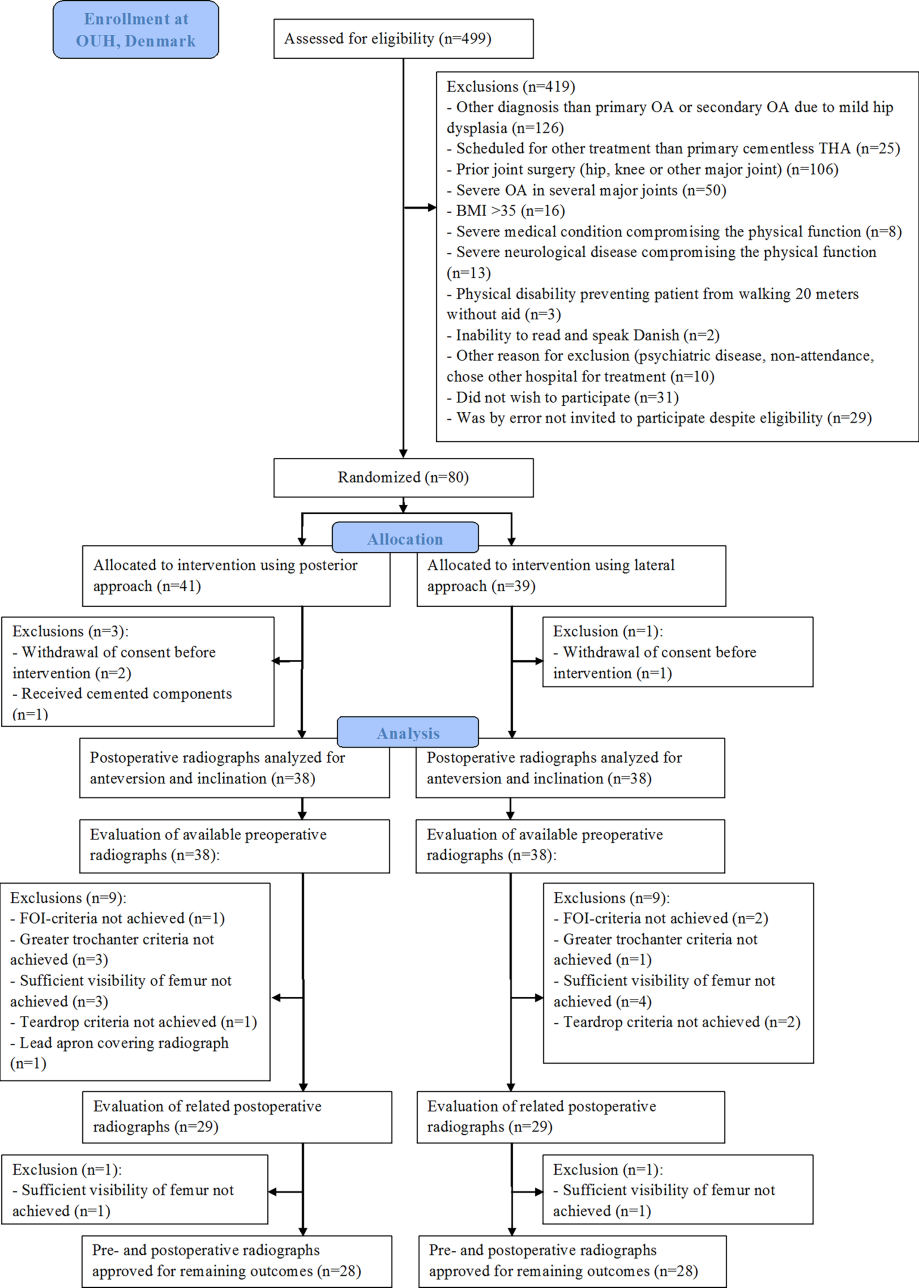
CONSORT flow diagram for enrolment of the patients according to inclusion and exclusion criteria and available radiographs. Please see text in ‘Participants’ and ‘Data collection’ for the trial’s inclusion criteria and the study’s radiographic inclusion criteria.

In total, 80 patients aged 45 to 70 years with unilateral primary hip osteoarthritis scheduled for cementless primary THA were recruited from the Department of Orthopaedic Surgery and Traumatology, Odense University Hospital, Denmark from May 2012 to May 2014 (Fig 1). Exclusion criteria are shown in Fig 1.

The trial complied with the Declaration of Helsinki and the Danish Data Protection Agency and was approved by The Danish Regional Committee on Biomedical Research Ethics (Southern Denmark) (project-ID S-20120009 and review number 42407). Informed written consent was obtained from all patients prior to inclusion.

The trial participants were assigned to surgery using PA or LA based on a random computer generated sequence, placed in sealed envelopes. This was handled by a nurse and a secretary, who did not take part in the patient recruitment or evaluation.

### Surgery

A surgeon from one of two teams, each consisting of three equally experienced surgeons performed the surgical intervention. The surgeons in each team performed only one of the two approaches. Prior to surgery on all patients, templating was performed in TraumaCad®. All patients were positioned in the lateral decubitus position during surgery and received the same type of cementless components (Bi-metric stem® and Exceed ABT Ringloc-x Shell™). All surgeons in both groups agreed upon a ‘target zone’ and aimed to position the cup within a ‘target zone’ of 15 ± 10° anteversion and 40 ± 10° inclination, as defined by Lewinnek et al [2]. The optimal position of the cup was not specified.

PA was performed using an incision over the posterior part of the greater trochanter through the fascia, followed by blunt dissection of the gluteus maximus muscle, then detachment of the external rotator muscles and incision of the posterior part of the hip capsule [19]. The hip was dislocated by internal rotation and flexion. During closure of the wound, capsular repair and re-insertion of the external rotators were performed followed by closure of the fascia, subcutaneous tissue and skin.

LA was performed using a midline incision over the greater trochanter and involved detachment of the anterior one-third of the gluteus medius insertion and gluteus minimus insertion on the greater trochanter. Excision of the capsule was performed on the anterior side of the joint, from the basis of the collum femoris to the acetabular rim. The hip was dislocated by external rotation, adduction and flexion. During closure of the wound, re-insertion of the muscles was performed. There was no capsular repair [20] and subcutaneous tissue and skin were closed with nylon suture to avoid visible suture clips on the postoperative radiographs as for PA.

Both groups received similar postoperative rehabilitation, which is described in detail elsewhere [17].

### Data collection

Preoperative and postoperative anterior-posterior radiographs of the pelvis were obtained according to a standardized protocol with the patient in a supine position and with the intention of having a 15° bilateral internal rotation of the hip joint with the center of the X-ray beam over the symphysis. All pictures were stored in Picture Archiving and Communication System (web-PACS), imported to TraumaCad® and assessed on the same type of high-resolution diagnostic screen (Fa. WIDE type 2103 CP) from June to September 2014. For calibration to actual bone size, we used a calibration marker ball (25.4mm) for the preoperative radiographs and the known size of the implanted prostheses head for the postoperative radiographs.

#### Radiographic inclusion criteria

To ensure sufficient quality of the radiographs pre-defined radiographic inclusion criteria were used. Thus all should have:

1. Tönnis foramen obturator index within 0.7–1.8 [21]
2. full visibility of several anatomical landmarks: a) greater and lesser trochanter on the hip of interest, b) both femurs 2.5 cm distal to lesser trochanter and c) detectable teardrops.

The foramen obturator index is a measure of pelvic rotation and calculated as the maximum horizontal width of the right obturator foramen divided by the width of the left obturator foramen [22]. If a patient’s preoperative radiograph was not included, the patient’s postoperative radiograph was evaluated only according to the foramen obturator index criteria and, if approved, cup anteversion and inclination were measured. Following the evaluation of the radiographs, 38 postoperative radiographs were included for the measurement of cup anteversion and inclination in each group and 28 pairs of preoperative and postoperative radiographs in each group were included for the measurement of femoral offset, cup offset, total offset, abductor moment arm, and leg length discrepancy (Fig 1).

#### Radiographic measurements

**Fig 2:**
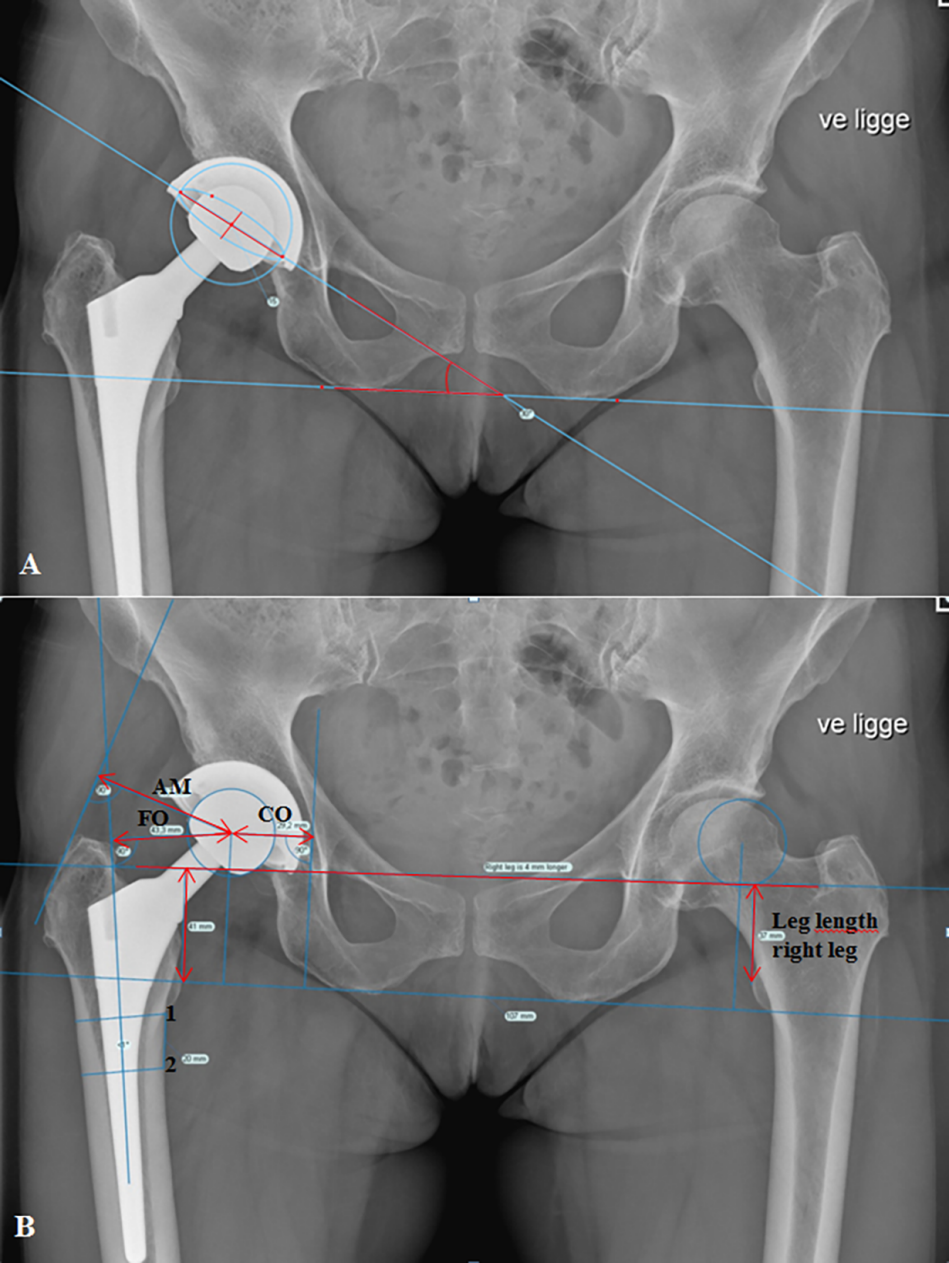
Example of measurements in TraumaCad®. (A) Anteversion and inclination on postoperative anterior-posterior radiographs of the pelvis. (B) Center of rotation (COR) marked on the operated side. The femoral shaft line was drawn from the center of two parallel lines with the shortest length across the femur extracted from two points marked at the distal level of the lesser trochanter and 20 mm distal from the lesser trochanter.

Cup anteversion and inclination were measured on postoperative radiographs (Fig 2A.). The definitions of these correspond with the radiographic definitions based on the coronal plane, as recommended by Wan et al. [23]. Measurements of femoral offset, cup offset, abductor moment arm and leg length discrepancy are shown in Fig 2B

Anteversion was measured using the option ‘Cup Version’ in TraumaCad®. ‘Cup Version* is a tool, where the user draws an ellipsis defining the opening rim of the cup. The opening rim of the implanted cup was defined by the long axis (line x) between the most superior and lateral point on the opening rim of the cup and the most inferior and medial point of opening rim of the cup. The short axis (line Y) was defined as the longest perpendicular line on line x measured from the anterior to the posterior opening rim of the cup. Based on that ellipsis, the program measured line x and line y and calculated the anteversion. Radiographic cup anteversion was defined as $arcsin\frac{short\;axis}{long\;axis}$ [2]. To verify cup anteversion the lateral view of the hip was assessed too. We found no retroverted cups in our study patients.

Radiographic cup inclination was measured as the angle of a horizontal line drawn between the two most inferior parts of both iscial tuberosities and a line drawn through the long axis of the ellipsis on the cup [2].

The secondary outcomes were measured on both preoperative and postoperative radiographs (Fig 2B).

Femoral offset was measured as the perpendicular distance from the center of rotation of the femoral head to the central axis of the femur [7, 9].

Cup offset was measured as the horizontal distance from the center of rotation to the vertical tangent of Koehler’s teardrop’s lateral side [1].

Total offset was calculated as the summation of femoral offset and cup offset.

Abductor moment arm was defined as the distance from the center of rotation to a perpendicular point on the tangent of the greater trochanter, representing the abductor muscle’s line of action [9, 10]. The tangent was drawn between two marking points, the most lateral point of the most superior part and the most inferior point of the most lateral point.

Leg length was measured as the length of a vertical line drawn from the most prominent point of the lesser trochanter perpendicular to a horizontal line drawn between the two acetabular teardrops [24].

#### Reliability

The protocol for all measurements was first practiced in a training session on 12 pairs of preoperative and postoperative radiographs. Then, a randomly selected sample of radiographs (n = 20) was assessed independently by three authors (CK, SR and LB, representing three levels of experience: inexperienced, experienced, very experienced) regarding the evaluation of inter-rater reliability. The same 20 patients were assessed twice by one author (CK) with a two-week interval for the evaluation of intra-rater reliability. We used two-way mixed-effects models with absolute agreement to calculate intra-class correlation coefficients (ICC). The average ICC value was reported for inter-rater reliability and the individual ICC value for intra-rater reliability. An ICC value of 0.00–0.20 represented slight reliability, 0.21–0.40 fair reliability, 0.41–0.60 moderate reliability, 0.61–0.80 substantial reliability and >0.81 almost perfect reliability [25]. Both intra-rater and inter-rater reliability showed almost perfect reliability for cup anteversion (ICC 1.00 and 0.93, respectively) and inclination (ICC 0.97 and 0.99, respectively). In supplement figures (S1 Fig and S2 Fig) we show the Bland-Altman-plots for the intra-rater reliability. They show a small measurement error of 2 degree in both anteversion and inclination. Similar strong reliabilities were shown for the secondary outcomes, except preoperative leg length discrepancy, which had an ICC score (0.77) representing substantial inter-rater reliability. The reason for this result remains unclear but is possibly related to discrepancies in identifying the single most prominent point on the lesser trochanter.

### Statistical analysis

A sample size was calculated for the main RCT but not for the specific outcomes of this explorative study. However, we performed a post hoc analysis and found a minimal detectable difference in anteversion of 4.1 degrees between the two groups, based on expected 35 patients in each group with the power level set to 80%. We used a two-sided power analysis for a two-sample means test, with an alpha level of 5%. Data were checked for normal distribution using the Shapiro-Wilk test and q-q plots and all data were found to be normally distributed. Mean, standard deviation (SD), mean difference between the two groups (LA and PA), 95% confidence interval (95% CI) and p-value were reported for each outcome. Unpaired two sample Student’s t-tests were used to test differences in continuous demographic data and outcome measures between the LA and PA groups. The chi-squared test was used to compare categorical demographic data and the number of cups placed inside or outside the ‘target zone’ in each group. The statistical analyses were performed using STATA version 13.1 (Stata Corp LP, Brownsville, TX, US) and with a 0.05 significance level.

### Demographics

There were no differences in patient characteristics between the two groups except that more patients in the LA group received a lateralized stem (Table 1).

**Table 1:** Patient characteristics.

Variable	Lateral approach (n = 38)	Posterior approach (n = 38)
Male	26	26
Age (years) mean (SD)	60.2 (45–69 years)	61.5 (47–69 years)
BMI (kg/m^2^) mean (SD)	26.9 (20–35)	27.6 (20–35)
Operated leg: right	20	19
32 mm head size 36 mm head size	30 8	32 6
Standard stem Lateralized stem	8 30	18 20

## Results

**Fig 3:**
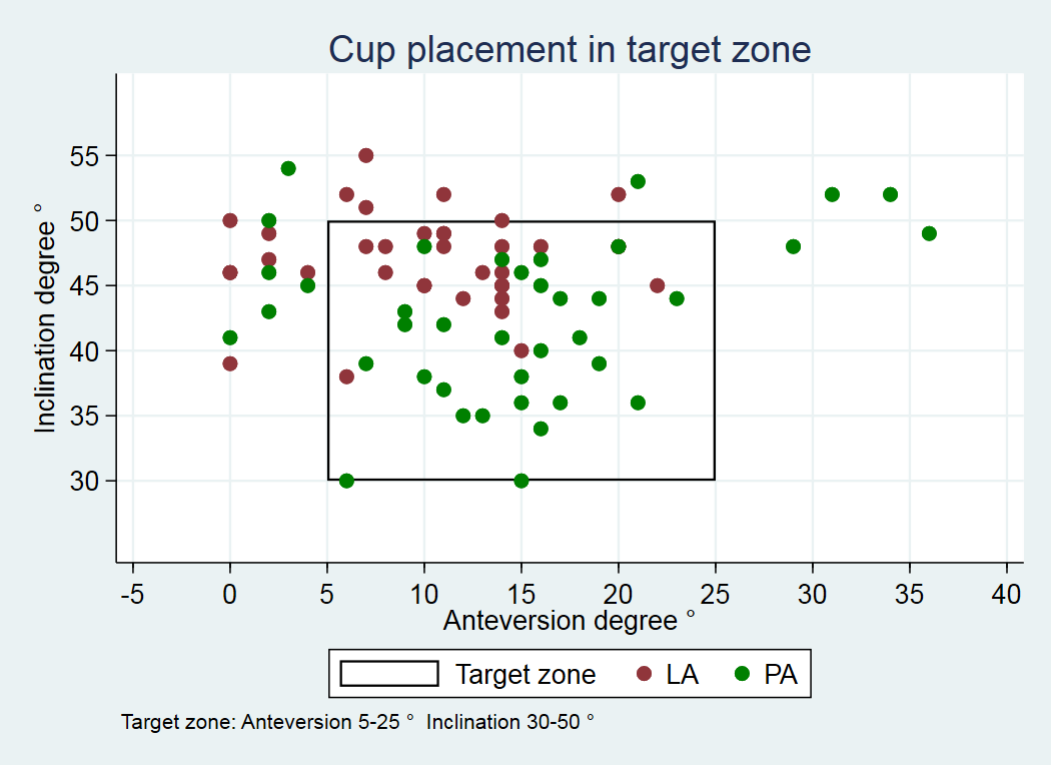
Cup position within/outside target zone. 28 cups from the PA group (green) and 25 cups from the LA group (red) lie within the ‘target zone’.

Anteversion was 5° (95% CI, -8.1 to -1.4; p = 0.006) larger in patients in the PA group than in the LA group, and inclination was 5° less steep (95% CI, 2.7 to 7.2; p<0.001) (Table 2). The number of cups placed outside the ‘target zone’ was equally distributed; 10 cups in the PA group and 13 cups in the LA group, with no statistically significant difference (p = 0.45) (Fig 3). However, only cups inserted using PA had an anteversion higher than the upper limit of the ‘target zone’.

**Table 2:** Cup position in the two groups.

Outcome	Lateral approach	Posterior Approach	Mean difference between groups (LA-PA)^†^
	Mean ±SD	Mean ±SD	Mean (95% CI), p-value
Anteversion* (°)	10 ± 5.9	15 ± 8.4	-5 (-8.1 to -1.4), 0.006
Inclination* (°)	47 ± 3.6	42 ± 5.9	5 (2.7 to 7.2), <0.001
ΔFO^#^ (mm)	0.8 ± 4.4	5.1 ± 6.6	-4.3 (-7.4 to -1.3), 0.006
ΔCO^#^ (mm)	-7.8 ± 3.8	-5.9 ± 4.3	-1.9 (-4.1 to 0.2), 0.08
ΔTO^#^ (mm)	-7.1 ± 5.6	-0.8 ± 6.8	-6.3 (-9.6 to -3), <0.001
ΔAM^#^ (mm)	-0.7 ± 5.4	4.1 ± 5.2	-4.8 (-7.6 to -1.9), 0.001
ΔLLD^#^(mm)	3.4 ± 5.0	1.9 ± 5.2	1.5 (-1.1 to 4.3), 0.3

*n = 38 in each group

^#^n = 28 in each group

^**†**^Tested with Student’s t-test with unequal variances in anteversion and inclination

Abbreviations: Δ = postoperative measurements subtracted from preoperative measurements. FO = femoral offset, CO = cup offset, TO = total offset, AM = abductor moment arm, LLD = leg length discrepancy.

The patients in the LA group had smaller change in femoral offset (95% CI, -7.4 to -1.3; p = 0.006), change in total offset (95% CI, -9.6 to -3; p<0.001) and change in abductor moment arm (95% CI, -7.6 to -1.9; p = 0.001) than patients in the PA group (Table 2). There were no statistically significant difference between the groups in change in cup offset (95% CI, -4.1 to 0.2; p = 0.08) and change in leg length discrepancy (95% CI, -1.1 to 4.3; p = 0.3).

## Discussion

This study showed significant differences in radiographic cup position between PA and LA following THA. This discovery may be a link to understanding the background for potential differences in clinical outcomes, such as dislocation rates, wear and hip abductor function.

However, data based on patient reported outcomes, which were the primary and secondary outcomes in the main trial [17], showed no significant difference between the groups except for self-reported limping [26].

The current study showed significantly more anteverted cups in the PA group than in the LA group and, thus, supported our first hypothesis. Furthermore, the inclination was significantly steeper in the LA group. The study by Callanan et al. 2011, based on evaluation of 1823 hips, showed the same difference in cup placement between the PA and LA as we found [12].

We found differences in cup placement despite the fact that the surgeons had agreed upon a ‘target zone’ for cup position prior to study start. These differences may be because the surgeons in the two teams aimed at a different ‘optimal’ cup position depending on the approach [27]. Only cups placed using PA were outside the ‘target zone’ in terms of high anteversion. This may be explained by the surgeon’s concern about posterior dislocation and as a consequence, anteversion of the cup may have been intentionally increased. In vivo studies have shown an association between high inclination, particularly inclinations ≥45°, and greater wear rates of the acetabular liner [5, 6, 28, 29].

The range for anteversion and inclination were narrower in the LA group than in the PA group. This supports the hypothesis that the view of the acetabulum is better using LA, which facilitates a more precise positioning of the cup compared with PA. Despite a statistically significant difference in cup position, both group means were within the ‘target zone’. The ‘target zone’ is highly debated and it has been shown that surgeons do not agree upon an optimal cup position [27]. Furthermore, no range of acetabular positioning has proven completely safe from dislocation, indicating that various factors influence stability of the hip [12, 30–33].

Choice of stem type and positioning of the cup can influence femoral offset and abductor moment arm [34]. A decreased femoral offset affects hip function and gait negatively [7, 35]. Femoral offset is positively correlated with abductor moment arm [9] and an increased femoral offset affects abductor strength positively and increases hip stability due to soft tissue tension [9, 10, 36]. The partial detachment of the gluteus medius tendon and the total detachment of the gluteus minimus muscle tendon from the greater trochanter during the LA procedure may affect the postoperative abductor muscle strength for this patient group [37–39]. Our study showed an increased femoral offset of 5.1mm and abductor moment arm of 4.1mm in the PA group, whereas the LA group showed unchanged femoral offset and abductor moment arm at 0,8mm and -0,7mm, respectively. We found a mean total offset postoperatively of -7.1 mm in patients in the LA group and -0.8 mm in the PA group (Table 2). This correlates well with data about hip muscle strength, where there has been shown poorer improvement in the LA group compared with the PA group in abductor muscle strength at 12 months follow up [39].

Sakalkale et al. concluded that a lateralized femoral component more closely restored hip biomechanics to the preoperative state [40]. In our study, more patients in the LA group received a lateralized stem and our results showed that femoral offset and abductor moment arm were held neutral, compared with the PA group. Cassidy et al. [7] found a similar tendency towards more use of lateralized stems in the unchanged femoral offset group. The use of lateralized stems in LA can be a result of the surgeon’s concern for the abductor muscle function postoperatively. The surgeon’s concern about dislocation following PA may lead to intentionally increased femoral offset and abductor moment arm to increase soft tissue tension, thus securing better joint stability. Another explanation for our findings may be a difference in stem anteversion between the two groups as an unknown confounder.

Some limitations of this study should be mentioned. First, the design of the study included randomization to two teams of three surgeons that only performed one approach each. Although, this prevented the results from being an evaluation of only one or two surgeon’s skill, learning curve or preferences for one approach. Second, stem anteversion was not measured due to lack of radiographs in lateral projections of both the femur and knee. The degree of stem anteversion could potentially influence the measurements of femoral offset and abductor moment arm. Third, Computed Tomography (CT) is considered the gold standard for the measurement of cup position, but Lewinnek’s methods for measurement of cup position on conventional radiographs have proven to be both valid and reliable compared with CT and we found in this study design an excellent intra-rater reliability [41, 42]. Fourth, the external validity may be compromised because about 1/4 of the patients included in the study had insufficient radiographs preoperatively, leaving a highly selected group in the analyses. However, this did not affect the primary analyses of cup position where only a few patients in each group were excluded. Finally, our study cannot contribute with knowledge about whether these observed radiographic differences have any clinical impact on hip function, wear rate or dislocation risk. We found a 5 degree difference in both anteversion and inclination between the groups. Based on the high intra-rater reliability and measurement error of 2 degree presented in Bland-Altmans plots (S1 Fig and S2 Fig), the difference seem real but most likely not clinically relevant.

## Conclusion

After total hip arthroplasty, cups inserted using the posterior approach had a larger anteversion but a less steep inclination when compared with cups inserted using the lateral approach. Statistically significant larger femoral offset, total offset and abductor moment arm were found in patients in the posterior approach group when compared with the lateral approach group. There was no statistically significant difference in cup offset and leg length discrepancy between the two surgical approaches.

## Supporting information

S1 File CONSORT check list. (DOC)

S2 File X-ray protocol. (DOCX)

S3 File Dataset. (XLSX)

S4 File Ethics committee_approval of additional protocol. (PDF)

S5 File Ethics committee_approval_Danish_main_study. (PDF)

S6 File Ethics committee_approval_English_main_study. (PDF)

S7 File Protokol_Ethics committee_version_3. (DOCX)

S1 Fig Blant-Altmann plot over intra-rater reliability for anteversion. (TIF)

S2 Fig Blant-Altmann plot over intra-rater reliability for inclination. (TIF)
