# Supplementary material for: Radiographic cup position following posterior and lateral approach to total hip arthroplasty. An explorative randomized controlled trial
Source: PLoS One. 2018 Jan 29;13(1):e0191401. doi: 10.1371/journal.pone.0191401 (PMC5788339; doi:10.1371/journal.pone.0191401)
Supplement: S2 File — (DOCX) [file pone.0191401.s002.docx]

**Protocol for**

**Radiographic cup position following posterior and lateral approach to total hip arthroplasty. An explorative randomized controlled trial.**

Christine Kruse^1^, Signe Rosenlund^1,2^, Leif Broeng^2^ and Søren Overgaard^1^

^1^Department of Orthopedic Surgery and Traumatology, Odense University Hospital, Institute of Clinical Research, University of Southern Denmark, Denmark
^2^Department of Orthopedic Surgery and Traumatology, Køge Hospital, Denmark

Keywords: Total hip arthroplasty, Cup placement, Osteoarthritis, Surgical approach
Clinical Trial registration: NCT01616667
Ethical Committee: S-20120009 and 42407

# Introduction

Many factors influence the outcome of the total hip arthroplasty (THA), both patient specific (e.g. diagnosis, weight and comorbidity (Hailer et al., 2012; Jolles et al., 2002; Nilsdotter et al., 2003)) and surgical specific factors (choice of prosthesis, the choice of surgical approach and placement of the components (Hailer et al., 2012; Jolles et al., 2002; Kennedy et al., 1998; Lewinnek et al., 1978; Lindgren et al., 2014; Masonis and Bourne, 2002)). Improper placement of components may be associated to dislocation, reduced abductor muscle strength and impingement (Biedermann et al., 2005; Cassidy et al., 2012; Dastane et al., 2011; Herman et al., 2011; Jolles et al., 2002; Lewinnek et al., 1978; McGrory et al., 1995; Patel et al., 2010; Ranawat et al., 2001; Romero et al., 2001) and could be related to the surgical approach. The two most common surgical approaches for THA are the posterior approach (PA) and the lateral approach (LA)(Jolles and Bogoch, 2006). Anteversion and inclination can be used to describe the acetabular cup orientation. A large anteversion and inclination increases the risk of dislocation (Biedermann et al., 2005). Lewinnek et al (Lewinnek et al., 1978) defined a “safe” zone of anteversion of 15 ± 10° and inclination of 40 ± 10°, within which the risk of dislocation is reduced. Several studies have found that PA is correlated to an increased risk of revision due to dislocation caused by instability compared to LA (Dudda et al., 2010; Hailer et al., 2012; Masonis and Bourne, 2002).

Placement of the components can also influence femoral offset (FO) and the abduction moment arm (AM) (Delp and Maloney, 1993). A decreased FO will affect hip function and gait negatively (Cassidy et al., 2012; Sariali et al., 2014). FO is positively correlated to AM and an increase affects the abductor strength positively and increases hip stability due to soft tissue tension (Brooks, 2013; McGrory et al., 1995; Romero et al., 2001). In addition to this, in LA the muscle function might be disturbed by partly detachment of the anterior part of the gluteus medius tendon and the total gluteus minimus muscle tendon from the greater trochanter, which rise concerns of postoperative function and pain (Amlie et al., 2014; Lindgren et al., 2014; Muller et al., 2011).

This randomized study will thus contribute with important reliable knowledge regarding the radiographic measurements of cup placement, femoral offset and abduction moment arm, comparing PA with LA.

## Aim

The aim of this study was to compare anteversion and inclination between the two surgical approaches as well as the change in measurements from preoperative to postoperative radiographs of femoral offset (FO), cup offset (CO), total offset (TO), abductor moment arm (AM) and leg length discrepancy. We additionally wanted to evaluate intra- and interobserver reliability of the methods used to measure the reported parameters.

We hypothesized that PA would have a larger anteversion and inclination and that LA would have a larger FO and AM postoperatively than PA. Furthermore, we hypothesized that we would have a high inter and intra reliability (ICC>0.81) of our measurements.

# Methods

## Design

This study is based on radiographic pre- and postoperatively explorative data from a randomized controlled trial, designed as a prospective, blinded, parallel-group, superiority trial with balanced randomization (1:1). The trial follows the CONSORT (Consolidated Standard of Reporting Trials) guidelines(Schulz et al., 2010), and is registered at ClinicalTrials.gov (registration no.: NCT01616667). A protocol has been published regarding the main study (Rosenlund et al., 2014).

## Participants

A total of eighty patients were recruited from the outpatient clinic at the Department of Orthopaedic Surgery and Traumatology, Odense University Hospital (OUH), Denmark between June 2012 and July 2014. The inclusion and exclusion criteria are listed in table 1. All patients were clinical and radiographically evaluated preoperatively and scheduled for THA. The trial complied with the Declaration of Helsinki. It was approved by the Danish Data Protection Agency and The Danish Regional Committee on Biomedical Research Ethics (Southern Denmark), project-ID S-20120009. A written and orally informed consent was collected prior to inclusion (Rosenlund et al., 2014).

| **Table 1.** Criteria for participants in the trial | |
| --- | --- |
| **Inclusion criteria** | **Exclusion criteria** |
| Age 45-70 year, both years included  Patients scheduled for primary cementless total hip arthroplasty  Endstage primary hip OA or secondary OA due to mild hip dysplasia (CE angle >20 degrees) | More joints (hip, knee or ankle) with expected joint arthroplasty within a year  Prior joint arthroplasty or any joints (hip, knee or ankle), or any joint related surgery on lower limbs, still providing symptoms  BMI > 35  Any physical disability preventing patients from walking 20 meters without aid  Any neurological disease (ex. Cerebral thrombosis, Parkinson) compromising the walking ability  Any severe medical condition compromising the physical function (ex. Chronic heart failure, chronic obstructive pulmonary disease). Evaluated by 30s-CST-test  Severe dementia (OMC <18)  Inability to read and understand Danish writing and oral instructions  Does not wish to participate |

## Randomization

The trial participants were assigned to either operation with THA through PA or through LA based on a random computer generated sequence, using sealed envelopes. The randomization procedure was handled by a nurse, who did not take part in the patient evaluation.

## Surgical procedure

The surgical intervention was performed by a surgeon from one of two teams of experienced specialist with special training in the given surgical approach. One team consisted of three surgeons, all with special training in LA, and the other team consisted of three surgeons, all with special training in PA. All patients received the same type of cementless components (Bi-metric stem® and Exceed ABT Ringloc-x Shell™) and the same care during hospital stay and rehabilitation.

During surgery, all patients were positioned in lateral decubitus position.
**Posterior approach:** PA was performed through a curved incision over the posterior part of greater trochanter through the fascia, followed by blunt dissection of gluteus maximus muscle. Then detachment of the external rotator muscles and incision of the posterior part of the hip capsule (Hoppenfeld S, 2009). The hip was dislocated by internal rotation and flexion. During closure, capsular repair and re-insertion of the external rotators were performed.
**Lateral approach:** LA was performed through a midline incision over the greater trochanter and involved detachment of the anterior one-third of the gluteus medius insertion and gluteus minimus insertion on the greater trochanter. Excision of the hip capsule was performed on the anterior side of the joint, from the basis of collum femoris to the acetabular rim. The hip was dislocated by external rotation, adduction and flexion. During closure of the wound, re-insertion of the detached part of the gluteus medius muscle and the gluteus minimus muscle was performed. There was no capsular repair (Mulliken et al., 1998).

Wounds were closed with nylon suture to avoid visible suture clips on the post-surgery radiographs. 10 cases (9 PA/1 LA) were accidentally closed with clips; however, it was not possible for the investigator (CK) a priori to determine the current approach on the postoperative radiographs.

## Radiographs

All radiographs were taken according to a standard protocol followed by the radiology department at OUH. Pre- and postoperative AP radiographs of the pelvis were obtained with the patient in supine position and with intention to have a 15 degrees bilateral internal rotation in the hip joint and centering of the X-ray beam over the symphysis pubis joint to avoid magnitude differences.

All radiographs were stored in Picture Archiving and Communication System (web-PACS) and imported to TraumaCad® for measurement of radiographic parameters. The preoperative pelvic radiographs were calibrated to actual bone size using the calibration marker ball (25.4mm). The postoperative radiograph was calibrated to actual bone size using the known size of the metal head of the prostheses implanted during surgery.

The radiographs were evaluated on the same type of high-resolution diagnostic screen (Fa. WIDE type 2103 CP).

## Radiographic inclusion criteria

To ensure sufficient quality of the radiographs we used the following pre-defined radiographic inclusion criteria: Tönnis foramen obturator index (FOI) within 0.7-1.8 (Jacobsen et al., 2004) and full visibility of the greater and lesser trochanter on the hip of interest as well as both femur 2.5 cm distal to the lesser trochanter and detectable radiographic teardrops. Tönnis’ FOI is a measure of pelvic rotation and was calculated as the maximum horizontal width of the right obturator foramen divided by the width of the left obturator foramen (Tonnis, 1976).

According to the above-mentioned criteria, 18 preoperative and 28 postoperative radiographs were excluded. 28 patients were invited to have a new postoperative radiograph taken (approved by The Danish Regional Committee on Biomedical Research Ethics on the 12^th^ of May 2014 (review number 42407)). All patients replied positively, but 4 patients were excluded because less than 2.5 cm of the femoral shaft was visible. If the preoperative radiograph was not approved, the patients’ postoperative radiographs were evaluated after the FOI-criteria only, and if approved, the anteversion and inclination were measured as the only parameters. The final number of patients in each group is shown in figure 1. The preoperative radiographs used for this study were taken with an average of 90 days (range, 0-424 days) before planned surgery. When the radiograph obtained within 3 months of the operation date was not approved according to the listed criteria, an older radiograph was used. The average time between operation date and postoperative radiograph were 139 days with a range of 2-668 days. The wide span was due to the new radiographs that were needed.


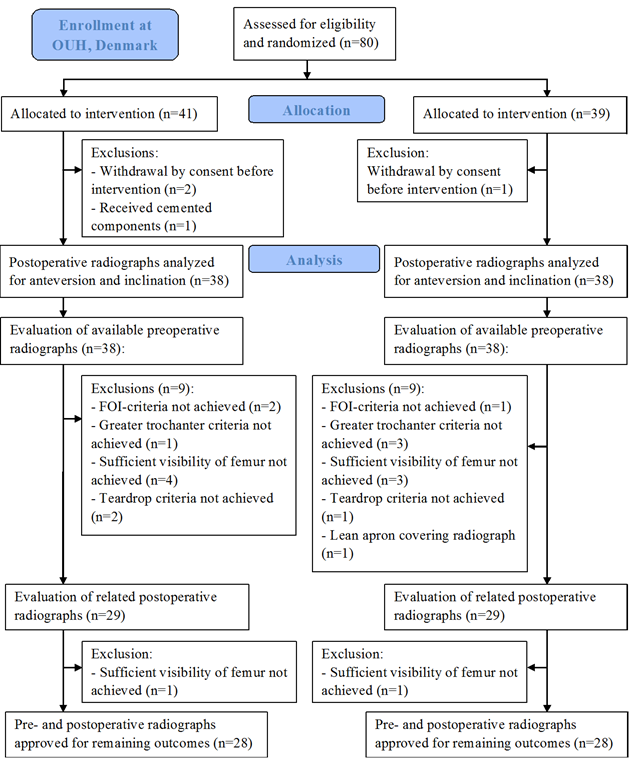


**Figure 1.** Flow chart of the recruitment inclusion and subsequent analysis of the patient’s radiographs.

## Data collection

Anteversion and inclination were measured on postoperative radiographs. The definition of anteversion and inclination used in this study were according to the radiographic definitions based on the coronal plane, as has been recommended by Wan et al. (Wan et al., 2009).

**Anteversion:** Radiographic anteversion was defined as $arcsin=\frac{short axis}{long axis}$ by Lewinnek et al. (Lewinnek et al., 1978). The long axis was the diameter of the implanted cup and the short axis the longest perpendicular line on the long axis measured from the anterior rim to the posterior rim of the cup. We used the option “Cup Version” in TraumaCad®, which is based on Lewinnek’s method (Fig. 2.A).

**Inclination:** The radiographic inclination was measured as the angle between a horizontal line drawn between the two most inferior parts of both iscial tuberosities and the line going through the long axis of the ellipse on the cup. The long axis was drawn through the most lateral and superior point and the most inferior and medial point of the cup (Fig. 2.A)

The following outcomes were measured on both pre- and postoperative radiographs (Fig. 2.B):

**Femoral offset:** Femoral offset (FO) was measured as the perpendicular distance from the center of rotation (COR) of the femoral head to the center axis of the femur (Cassidy et al., 2012; Herman et al., 2011; Krishnan et al., 2006; McGrory et al., 1995).

**Cup offset:** Cup offset (CO) was measured as the horizontal distance from the COR to the vertical tangent of Koehler’s teardrop’s lateral side (Jolles et al., 2002).

**Total offset:** Total offset (TO) was calculated as the summation of FO and CO.

**Abduction moment arm:** Abduction moment arm (AM) is the length of a straight line from the COR of the femoral head to a perpendicular point of the tangent of the greater trochanter, representing the abductor muscle’s line of action (McGrory et al., 1995; Romero et al., 2001). The tangent of greater trochanter was drawn between to marking spots, the most lateral spot of the most superior part and the most inferior spot of the most lateral point.

**Leg length:** The standard method for measuring leg length as described by Ranawat et al (Ranawat et al., 2001) was used. A horizontal line was drawn between the two acetabular teardrops and two perpendicular vertical lines that represented the leg length, was drawn between the most prominent points on the lesser trochanters and the trans-teardrop line.


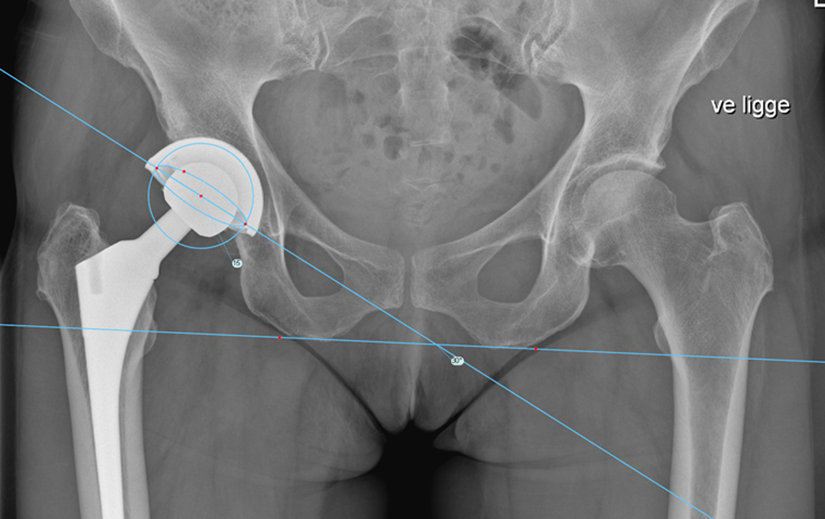


**A**

**
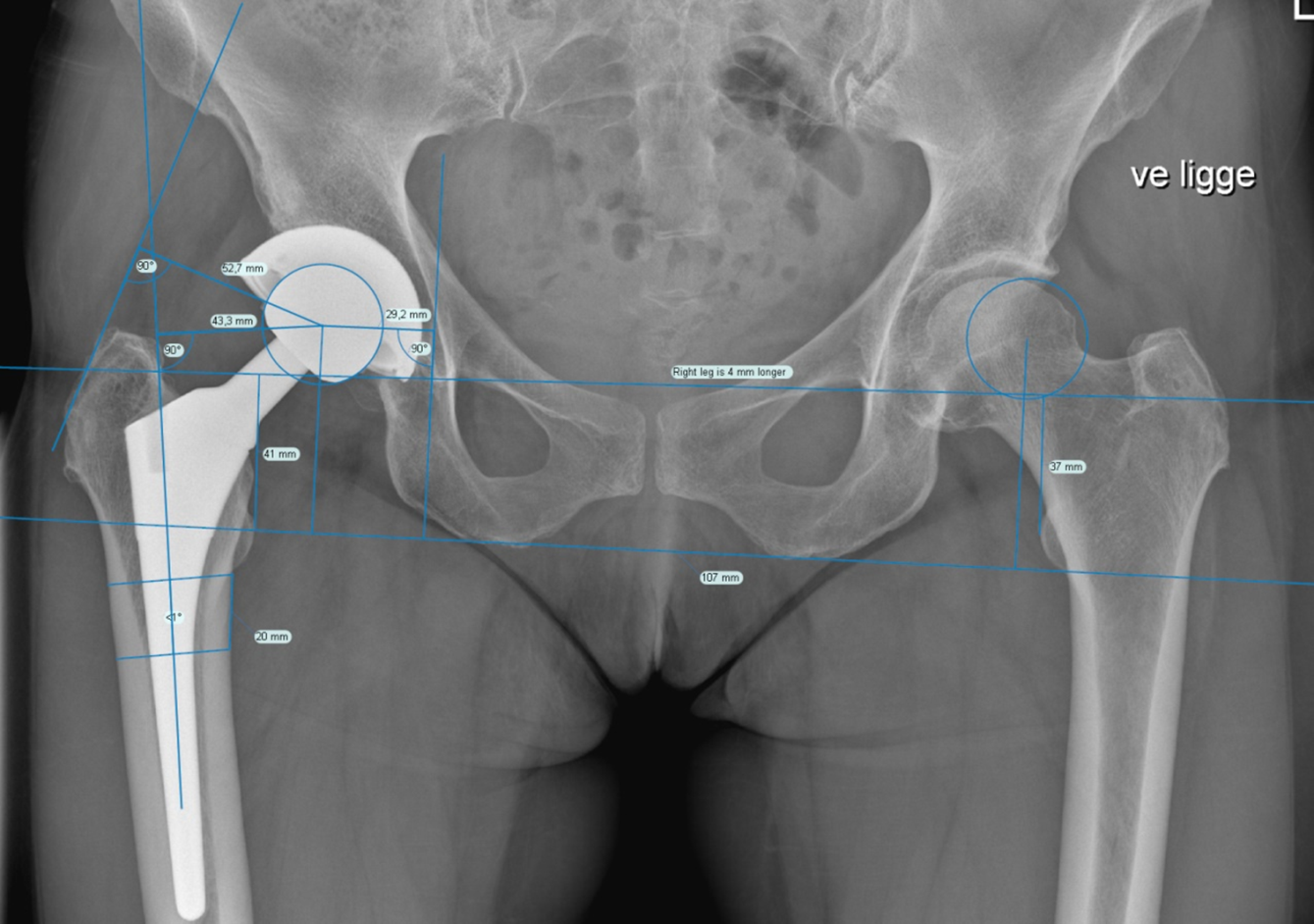
**

**B**

**Figure 2.** Radiographic example of measurements set in TraumaCad®: **(A)** Anteversion and inclination postoperative AP radiographs of the pelvis. **(B)** Center of rotation (COR), the center of the circled – marked on the operated side. The femoral shaft line was drawn from the center of two parallel lines extracted from two spots marked from the distal level of trochanter minor and 20 mm distal from the lesser trochanter and drawn across the femur indicating the shortest width. Femoral offset (43.3mm), cup offset (29.2mm), abductor moment arm (52.7mm) and leg length discrepancy (4mm) were measured according to methods described.

**Intra- and interobserver reliability:** All outcomes were measured by one author (CK), blinded for the randomization sequence. A random selected sample-size of 20 patients had all outcomes measured by three authors (CK, SR and LB) independently regarding the evaluation of interobserver reliability, and the 20 patients was measured twice by the same author (CK) with a two weeks interval for the evaluation of intraobserver reliability.

## Statistics

Data was checked for normal distribution and treated accordingly. Data are reported with mean and standard deviation for each outcome in the two groups (PA and LA). An unpaired t-test was used as significant test when testing for differences in outcomes between the two groups. The chi squared test was used for comparing the number of cups placed outside the safe zone in each group.

Intraclass correlation coefficient (ICC) and confidence interval was calculated to assess the intra- and interobserver reliability. To calculate the interobserver reliability, we used a two-way mixed-effects model with absolute agreement, where the patients were the random effects (n=20) and the observers were the fixed effects (n=3), reporting the average ICC value. Calculating intraobserver reliability, we used a two-way mixed-effects model with absolute agreement, where the patients were the random effects (n=20) and the observers were the fixed effect (n=2), reporting the individual ICC value. An ICC value >0.81 is considered excellent strength of agreement (Landis and Koch, 1977).

The statistical significance level was set at p<0.05. The statistics was performed using STATA version 13.1 (StataCorp LP, TX, USA).

# References

Amlie, E., Havelin, L.I., Furnes, O., Baste, V., Nordsletten, L., Hovik, O. and Dimmen, S., 2014. Worse patient-reported outcome after lateral approach than after anterior and posterolateral approach in primary hip arthroplasty. Acta Orthop, 85(5): 463-9.

Biedermann, R., Tonin, A., Krismer, M., Rachbauer, F., Eibl, G. and Stockl, B., 2005. Reducing the risk of dislocation after total hip arthroplasty: the effect of orientation of the acetabular component. J Bone Joint Surg Br, 87(6): 762-9.

Brooks, P.J., 2013. Dislocation following total hip replacement: causes and cures. Bone Joint J, 95-b(11 Suppl A): 67-9.

Cassidy, K.A., Noticewala, M.S., Macaulay, W., Lee, J.H. and Geller, J.A., 2012. Effect of femoral offset on pain and function after total hip arthroplasty. J Arthroplasty, 27(10): 1863-9.

Dastane, M., Dorr, L.D., Tarwala, R. and Wan, Z., 2011. Hip offset in total hip arthroplasty: quantitative measurement with navigation. Clin Orthop Relat Res, 469(2): 429-36.

Delp, S.L. and Maloney, W., 1993. Effects of hip center location on the moment-generating capacity of the muscles. J Biomech, 26(4-5): 485-99.

Dudda, M., Gueleryuez, A., Gautier, E., Busato, A. and Roeder, C., 2010. Risk factors for early dislocation after total hip arthroplasty: a matched case-control study. J Orthop Surg (Hong Kong), 18(2): 179-83.

Hailer, N.P., Weiss, R.J., Stark, A. and Karrholm, J., 2012. The risk of revision due to dislocation after total hip arthroplasty depends on surgical approach, femoral head size, sex, and primary diagnosis. An analysis of 78,098 operations in the Swedish Hip Arthroplasty Register. Acta Orthop, 83(5): 442-8.

Herman, K.A., Highcock, A.J., Moorehead, J.D. and Scott, S.J., 2011. A comparison of leg length and femoral offset discrepancies in hip resurfacing, large head metal-on- metal and conventional total hip replacement: a case series. J Orthop Surg Res, 6(65): 65.

Hoppenfeld S, D.P., Buckley R, 2009. The Hip. Surgical Exposures in Orthopaedics. The Anatomic Approach, 4th edition Lippinoctt Williams & Wilkins: 403-462.

Jacobsen, S., Sonne-Holm, S., Lund, B., Soballe, K., Kiaer, T., Rovsing, H. and Monrad, H., 2004. Pelvic orientation and assessment of hip dysplasia in adults. Acta Orthop Scand, 75(6): 721-9.

Jolles, B., Zangger, P. and Leyvraz, P., 2002. Factors predisposing to dislocation after primary total hip arthroplasty: a multivariate analysis. J Arthroplasty, 17(3): 282-8.

Jolles, B.M. and Bogoch, E.R., 2006. Posterior versus lateral surgical approach for total hip arthroplasty in adults with osteoarthritis. Cochrane Database Syst Rev(3): Cd003828.

Kennedy, J., Rogers, W., Soffe, K., Sullivan, R., Griffen, D. and Sheehan, L., 1998. Effect of acetabular component orientation on recurrent dislocation, pelvic osteolysis, polyethylene wear, and component migration. J Arthroplasty, 13(5): 530-4.

Krishnan, S.P., Carrington, R.W., Mohiyaddin, S. and Garlick, N., 2006. Common misconceptions of normal hip joint relations on pelvic radiographs. J Arthroplasty, 21(3): 409-12.

Landis, J.R. and Koch, G.G., 1977. The measurement of observer agreement for categorical data. Biometrics, 33(1): 159-74.

Lewinnek, G.E., Lewis, J.L., Tarr, R., Compere, C.L. and Zimmerman, J.R., 1978. Dislocations after total hip-replacement arthroplasties. J Bone Joint Surg Am, 60(2): 217-20.

Lindgren, J.V., Wretenberg, P., Karrholm, J., Garellick, G. and Rolfson, O., 2014. Patient-reported outcome is influenced by surgical approach in total hip replacement: a study of the Swedish Hip Arthroplasty Register including 42,233 patients. Bone Joint J, 96-B(5): 590-6.

Masonis, J.L. and Bourne, R.B., 2002. Surgical approach, abductor function, and total hip arthroplasty dislocation. Clin Orthop Relat Res(405): 46-53.

McGrory, B.J., Morrey, B.F., Cahalan, T.D., An, K.N. and Cabanela, M.E., 1995. Effect of femoral offset on range of motion and abductor muscle strength after total hip arthroplasty. J Bone Joint Surg Br, 77(6): 865-9.

Muller, M., Tohtz, S., Springer, I., Dewey, M. and Perka, C., 2011. Randomized controlled trial of abductor muscle damage in relation to the surgical approach for primary total hip replacement: minimally invasive anterolateral versus modified direct lateral approach. Arch Orthop Trauma Surg, 131(2): 179-89.

Mulliken, B.D., Rorabeck, C.H., Bourne, R.B. and Nayak, N., 1998. A modified direct lateral approach in total hip arthroplasty: a comprehensive review. J Arthroplasty, 13(7): 737-47.

Nilsdotter, A.K., Petersson, I.F., Roos, E.M. and Lohmander, L.S., 2003. Predictors of patient relevant outcome after total hip replacement for osteoarthritis: a prospective study. Ann Rheum Dis, 62(10): 923-30.

Patel, A., Wagle, R., Usrey, M., Thompson, M., Incavo, S. and Noble, P., 2010. Guidelines for implant placement to minimize impingement during activities of daily living after total hip arthroplasty. J Arthroplasty, 25(8): 1275-81.e1.

Ranawat, C.S., Rao, R.R., Rodriguez, J.A. and Bhende, H.S., 2001. Correction of limb-length inequality during total hip arthroplasty. J Arthroplasty, 16(6): 715-20.

Romero, A.C., Imrie, S. and Goodman, S.B., 2001. Sliding trochanteric osteotomy preserves favorable abductor biomechanics in revision total hip arthroplasty. J Arthroplasty, 16(1): 55-64.

Rosenlund, S., Broeng, L., Jensen, C., Holsgaard-Larsen, A. and Overgaard, S., 2014. The effect of posterior and lateral approach on patient-reported outcome measures and physical function in patients with osteoarthritis, undergoing total hip replacement: A randomised controlled trial protocol. BMC Musculoskelet Disord.

Sariali, E., Klouche, S., Mouttet, A. and Pascal-Moussellard, H., 2014. The effect of femoral offset modification on gait after total hip arthroplasty. Acta Orthop, 85(2): 123-7.

Schulz, K.F., Altman, D.G. and Moher, D., 2010. CONSORT 2010 statement: updated guidelines for reporting parallel group randomised trials. Bmj, 340: c332.

Tonnis, D., 1976. Normal values of the hip joint for the evaluation of X-rays in children and adults. Clin Orthop Relat Res(119): 39-47.

Wan, Z., Malik, A., Jaramaz, B., Chao, L. and Dorr, L.D., 2009. Imaging and navigation measurement of acetabular component position in THA. Clin Orthop Relat Res, 467(1): 32-42.
