# Supplementary material for: Radiographic cup position following posterior and lateral approach to total hip arthroplasty. An explorative randomized controlled trial
Source: PLoS One. 2018 Jan 29;13(1):e0191401. doi: 10.1371/journal.pone.0191401 (PMC5788339; doi:10.1371/journal.pone.0191401)
Supplement: S4 File — (PDF) [file pone.0191401.s004.pdf]

De Videnskabsetiske Komitéer
for Region Syddanmark

komite@rsyd.dk

Læge
Signe Rosenlund
Køge Sygehus
Ortopædkirurgisk afdeling H
Lykkebækvej 1
4600 Køge

13. maj 2014

Projekt-ID: S-20120009
HLP

Vedr. forskningsprojekt: Undersøgelse af to kirurgiske adgange ved indsættelse af primær total hoftealloplastik hos slidgigtspatienter. Effekt på den tidlige fysiske funktion, smerter, patientrapporterede resultater, gangmønster og muskelstyrke. - et klinisk randomiseret forsøg

Den Videnskabsetiske Komité for Region Syddanmark har den 31. marts 2014 modtaget en anmeldelse af tillæg med anmeldelses nr. 42407, dateret 31. marts 2014.

Tillæg nr. 1 omhandler:
Som led i det oprindelige studie 3 vil vi gerne vurdere placeringen af protesekomponenterne (inklination, anteversion, femur- offset, cup-offset, abduktor moment og benlængde). Protesekomponenternes placering kan have betydning for risikoen for om hoften går af led og om protesen bliver slidt eller sætter sig løs med tiden. Dette kan have betydning for den samlede succes af operationen. Placering af protesekomponenterne kan variere afhængig af hvilken kirurgisk adgang der benyttes, da de to forskellige metoder giver forskellig visuel præsentation af hhv. hofteledskålen og lårbenet. Dette kan påvirke kirurgens orientering og derved også placering af protesen. Proteseplaceringen er ikke tidligere undersøgt i et RCT studie, som dette projekt giver mulighed for, hvorfor studiet vil bidrage med væsentlig ny viden omkring de to kirurgiske adgange.
I forbindelse med røntgenbilledestudiet har vi opsat kriterier til billedkvaliteten, for at muliggøre valide målinger. Det er nødvendigt at kunne se den øverste del af lårbenet og bækkenet ikke må være roteret.
Alle patienter får rutinemæssigt taget post-operative billeder efter en fastlagt protokol fra røntgenafdelingens side, hvilket burde sikre en tilstrækkelig kvalitet af billederne.
Vi har nu gennemgået vores datamateriale og har konstateret at 24 patienters billeder ikke lever op til denne kvalitet. For at vi kan gennemfører en fornuftig analyse af proteseplaceringen mellem de to kirurgiske adgange er det vigtigt at genindkalde disse patienter og tage et nyt billede af bækkenet, som lever op til kravene.

Komiteen har ingen yderligere bemærkninger til det fremsendte materiale og kan godkende ovenstående tillæg.

Godkendelsen omfatter følgende dokumenter:

- Underskrevet anmeldelse af tillæg, dateret 31. marts 2014.
- Deltagerinformation røntgen version 6.0, modtaget 12. maj 2014.
- Samtykkeerklæring version S2, dateret, modtaget 7. maj 2014.
- E-mail fra Signe Rosenlund, modtaget 12. maj 2014.

Sagen har været behandlet den 28. april samt færdigbehandlet og godkendt den 12. maj af Komité 2's formand, professor, overlæge dr. Med., Jens Michael Hertz.

Venlig hilsen

Heidi Lund Pedersen
AC-Fuldmægtig

Kopi til: Professor, overlæge, Søren Overgaard,
Odense Universitets Hospital, soeren.overgaard@rsyd.dk
