# Supplementary material for: Radiographic cup position following posterior and lateral approach to total hip arthroplasty. An explorative randomized controlled trial
Source: PLoS One. 2018 Jan 29;13(1):e0191401. doi: 10.1371/journal.pone.0191401 (PMC5788339; doi:10.1371/journal.pone.0191401)
Supplement: S5 File — (PDF) [file pone.0191401.s005.pdf]

Læge
Signe Rosenlund
Køge Sygehus
Ortopædkirurgisk afdeling H
Lykkebækvej 1
4600 Køge

Den Videnskabsetiske Komité for Region Syddanmark

komite@regionsyddanmark.dk

21. marts 2012

Projekt-ID: S-20120009
CKH/csf

Forskningsprojekt: Undersøgelse af to kirurgiske adgange ved indsættelse af primær total hoftealloplastik hos slidgigtspatienter. Effekt på den tidlige fysiske funktion, smerter, patientrapporterede resultater, gangmønster og muskelstyrke. - et klinisk randomiseret forsøg

Den Videnskabsetiske Komité for Region Syddanmark har den 9. marts 2012 modtaget revideret materiale vedrørende forskningsprojektet.

Afgørelse
Komiteen kan herefter godkende forskningsprojektet i henhold til lov nr. 402 af 28. maj 2003.

Godkendelsen gælder for de anmeldte forsøgssteder, de anmeldte forsøgsansvarlige i Danmark samt for den angivne forsøgsperiode. Komiteen antager, at den forsøgsansvarlige drager omsorg for at underrette de øvrige deltagere i projektet om Komiteens afgørelse i sagen.

Godkendelsen gælder til den 31. januar 2015 og følgende dokumenter er lagt til grund ved vurderingen:

- Underskrevet anmeldelse dateret den 12. december 2012
- Protokol version 3
- Deltagerinformation version 4 af 9. marts 2012
- Deltagerinformation til raske forsøgspersoner version 3 af 9. marts 2012
- Samtykkeerklæring af 12. december 2011
- Lægmandsresume version 2
- Opslag modtaget 12. december 2011
- Spørgeskemaer modtaget 12. december 2012

Iværksættelse af projektet i strid med godkendelse kan straffes med bøde eller fængsel, jf. komitelovens § 29.

Ændringer
Foretages der væsentlige ændringer i protokolmaterialet under gennemførelsen af projektet, skal disse anmeldes til Komitéen i form af tillægsprotokoller. Ændringerne må først iværksættes efter godkendelse fra Komitéen, jf. komitélovens § 23, stk. 1, nr. 1.

Anmeldelse af tillægsprotokoller skal ske elektronisk på www.drvk.dk med det allerede tildelte anmeldelsesnummer og adgangskode.
Væsentlige ændringer er bl.a. ændringer, der kan få betydning for forsøgspersonernes sikkerhed, fortolkning af den videnskabelige dokumentation, som projektet bygger på, samt gennemførelsen eller ledelsen af projektet. Det kan f. eks. være ændringer i in- og eksklusionskriterier, forsøgsdesign, antal forsøgspersoner, forsøgsprocedurer, behandlingsvarighed, effektparametre, ændringer om de forsøgsansvarlige eller forsøgssteder samt indholdsmæssige ændringer i det skriftlige informationsmateriale til forsøgspersonerne.

Hvor nye oplysninger betyder, at forskeren overvejer at ændre proceduren eller stoppe forsøget, skal Komitéen orienteres om det.

Bivirkninger og hændelser
Der gøres opmærksom på, at den forsøgsansvarlige omgående skal indberette til Komitéen, hvis der optræder alvorlige bivirkninger eller hændelser.

Én gang årligt i hele forsøgsperioden skal Komitéen have tilsendt en liste over alle alvorlige bivirkninger og alvorlige hændelser, som er indtruffet i forsøgsperioden sammen med en rapport om forsøgspersonernes sikkerhed, jf. komitélovens § 22, stk. 4.

Materialet skal være på dansk. Listen over alvorlige bivirkninger og alvorlige hændelser kan dog være på engelsk, hvis der er vedlagt et dansk resumé.

Afslutning
I henhold til komitéloven skal den forsøgsansvarlige senest 90 dage efter afslutningen af forskningsprojektet underrette Komitéen om afslutningen af projektet.

Afbrydes projektet tidligere end planlagt, skal en begrundelse herfor sendes til Komiteen senest 15 dage efter, at beslutningen er truffet, jf. komitélovens § 22, stk. 5.

Hvis projektet ikke påbegyndes, skal dette samt årsagen hertil meddeles Komiteen.

Komiteen beder om kopi af den afsluttende forskningsrapport eller publikation, jf. komitélovens § 22, stk. 2. Vi skal i den forbindelse gøre opmærksom på, at der er pligt til at offentliggøre såvel negative som positive forsøgsresultater, jf. komitélovens § 14, stk. 1, nr. 6

Projekt-ID bedes anført ved fremsendelsen. Henvendelser vedrørende projektet kan rettes til Komitéens sekretariat.

Tilsyn
Komiteen fører tilsyn med, at projektet udføres i overensstemmelse med godkendelsen, jf. komitélovens § 22, stk. 1.

Følgende komitémedlemmer deltog i mødebehandlingen den 23. januar 2012:

- Birger Møller
- Henrik Steen Hansen
- Jeppe Gram
- Lone Agertoft
- Kirsten Kyvik
- Freddie H. Madsen
- Sonny Berthold
- Susanne Linnet
- Claus Warming
- Jette Jensen

Sagen har været behandlet og endelig godkendt af Komitéens formand, overlæge, dr. med. Birger Møller den 20. marts 2012.

På Komiteens vegne,
venlig hilsen

Birger Møller
Formand

/

Claus Kvist Hansen
Sekretariatsleder

Kopi til: Professor, overlæge, Søren Overgaard, Odense Universitets Hospital, Ortopædkirurgisk afdeling O, Sdr. Boulevard 29, 5000 Odense C.
