# Supplementary material for: Radiographic cup position following posterior and lateral approach to total hip arthroplasty. An explorative randomized controlled trial
Source: PLoS One. 2018 Jan 29;13(1):e0191401. doi: 10.1371/journal.pone.0191401 (PMC5788339; doi:10.1371/journal.pone.0191401)
Supplement: S6 File — (PDF) [file pone.0191401.s006.pdf]

Dr. Med.
Signe Rosenlund
Køge Sygehus
Ortopædkirurgisk afdeling H
Lykkebækvej 1
4600 Køge.

The Regional Scientific Ethical Committee for Southern Denmark

komite@regionsyddanmark.dk

9. august 2013

Projekt-ID: S-20120009
HLP

Research project: The Effect of Posterior and Lateral Approach on Patient-Reported Outcome Measures and Physical Function in Patients with Osteoarthritis, Undergoing Total Hip Replacement. A Randomized Controlled Trial

The Committee on Biomedical Research Ethics for the Southern Region of Denmark hereby certify, that the above mentioned research project has been approved, and is registered to take place in the period 01-02-2012 until 31-01-2015

Generally, an approval is valid throughout the accepted project period, and a regular confirmation of the approval does not take place. Unless otherwise stated, the approval includes all documents written in Danish presented to the Committee up until the date of approval. A project can under certain circumstances be prolonged by an additional application to the Committee.

According to Danish law, all biomedical research taking place in Denmark must be approved by the Committees and comply with Danish legislation (Law no. 503 of June 24th 1992, no. 1133 of December 21st 1994, no. 499 of June 12th 1996, no. 402 of May 28th 2003, and no. 272 of April 1st 2006). Please note, that "GCP" and "ICH-GCP" rules are only partially implemented in Danish law. Additionally, the Committees only approve the part of a project that is written in Danish, and statistical passages written in English.

The Committees never undertake the task of investigating whether a project meets the requirements of foreign laws and legislation, and does not confirm accordance in contents between translated projects.

Further inquiries can be addressed to the Committee Secretariat.

Yours Sincerely,

Heidi Lund Pedersen
Academic, Administrative Officer

Regionshuset, Damhaven 12, 7100 Vejle, Denmark
Phone.:(+45) 76638220 – 76638221 – 76638222
Homepage: www.regionsyddanmark.dk/komit
