# Supplementary material for: Radiographic cup position following posterior and lateral approach to total hip arthroplasty. An explorative randomized controlled trial
Source: PLoS One. 2018 Jan 29;13(1):e0191401. doi: 10.1371/journal.pone.0191401 (PMC5788339; doi:10.1371/journal.pone.0191401)
Supplement: S7 File — (DOCX) [file pone.0191401.s007.docx]

**Undersøgelse af to kirurgiske adgange**

**ved indsættelse af primær total hoftealloplastik**

**hos slidgigtspatienter.**

**Effekt på den tidlige fysiske funktion, smerter,**

**patientrapporterede resultater, gangmønster og**

**muskelstyrke**

**- et klinisk randomiseret forsøg**

Protokol til Ph.d.-studie ved

Læge Signe Rosenlund

December 2011

Syddansk Universitet

Vejledere

Søren Overgaard, Professor, overlæge, Ortopædkirurgisk afdeling O, Odense Universitets Hospital

Leif Broeng, Overlæge, Ortopædkirurgisk afdeling H, Køge Sygehus, Lykkebækvej 1, 4600 Køge

Anders Holsgaard Larsen, cand.scient, lektor, Klinisk Institut, Syddansk Universitet, Sdr. Boulevard 29, 5000 Odense C

Carsten Jensen, cand.scient., PhD., Klinisk Institut, Syddansk Universitet, Sdr. Boulevard 29
5000 Odense C

# 1.0 Indledning

Indsættelse af en kunstig hofte, hvor både hofteledskål og hofteledhovedet skiftes kaldes også primær total hoftealloplastik (THA), og er blevet et af ​​de hyppigste og mest succesfulde ortopædkirurgiske indgreb i dag^1-3^. I Danmark udføres næsten 10.000 primære operationer og 1.600 om-operationer hvert år. Antallet af operationer forventes at stige, pga. den voksende ældrebefolkning^1^. Både i Danmark og internationalt er de hyppigste anvendte kirurgiske adgange, den bagerste adgang (posterior adgang=PA) og sideadgang (laterale adgang =LA) ^1, 4-6^. Ved kirurgisk adgang forstås den måde hvorpå kirurgen skaber adgang til hofteleddet. I Danmark er 91% af alle THA’er indsat ved hjælp af PA og kun 8% er indsat ved hjælp af LA. I Sverige og Norge er billedet noget anderledes, her tegner PA sig for henholdsvis 60% og 24%, mens LA tegner sig for henholdsvis 40% og 75% ^7^. I litteraturen beskrives hver adgang med fordele og ulemper. LA, som bl.a. indbefatter overskæring af muskelstrukturer på siden af hofteleddet, bliver ofte forbundet med ringere fysisk funktionsniveau, flere postoperative hoftesmerter, ændret gangmøsnter/halten og reduceret hofte-muskelstyrke. PA er forbundet med en højere rate af proteseledskred (ledhovedet skrider ud af ledskålen), på grund af beskadigelse af de bagerste strukturer omkring hofteleddet^7^. Ved gennemgang af litteraturen findes ingen konsensus om, hvilken operationsmetode der er bedst^4-6, 8, 9^. De eksisterende studier er ofte utilstrækkelige, idet de er af ældre dato, med få patienter involveret og de opfylder ikke altid de krav, der stilles til god kliniks forskning^4^.

Proteseledskred er en af ​​de vigtigste årsager til om-operation efter primær THA, og tegner sig for 34% af alle årsager til om-operation i Danmark. I Sverige og Norge udgør proteseledskred som indikation for om-operation kun 23%^7^. En norsk undersøgelse har desuden vist, at proteser indsat via LA har en længere 10 års protese-overlevelse end proteser indsat via PA, hvor om-operation er slut-punkt^8^. Den lavere overlevelsesrate i PA-gruppen kan forklares ved den øgede forekomst af om-operationer på grund af proteseledskred^8^. Proteseledskred er en meget ubehagelig tilstand for den enkelte patient, da det giver voldsomme smerter i hoften og fuldstændig manglende evne til at støtte på benet, så mange oplever at falde i forbindelse med at protesen går af led. Tilstanden kræver indlæggelse på hospitalet og ofte en kortvarig bedøvelse, hvor hofteprotesen trækkes på plads.

Proteseoverlevelsen og om-operationsraten beskriver dog ikke fuldt ud succesen med THA, da sub-optimale resultater med f.eks. kroniske hoftesmerter eller nedsat fysisk funktionsniveau ikke nødvendigvis fører til om-operation^10^. Patient-rapporterede effektmål (PROM=patient reported outcome meassures, indsamles vha. spørgeskemaer) bliver ofte brugt som evalueringsmetode af primære THA med henblik på at nuancere den samlede viden på området^11-16^. Der er generel enighed om, at PROM forbedres markant efter udførelse af THA hos slidgigtspatienter. Vi har kun kendskab til to ikke-randomiserede undersøgelser af PROM mellem PA og LA^5, 6^. Begge studier fandt betydelige forskelle i henhold til både smerte og fysisk funktionsniveau, hvor LA havde de signifikant dårligste resultater. En dansk undersøgelse har vist, at kroniske smerter er til stede hos 28% af alle patienter efter primær THA, uden hensyntagen til hvilken kirurgisk adgang, der blev brugt^17^. 12% af patienterne oplyste, at smerterne påvirkede deres dagligdag. Undersøgelsen fandt også at patienternes indrapportering om graden af kroniske smerter var forbundet med patienternes indrapportering af intensiteten af ​​de akutte postoperative smerter^17^. Der findes ingen undersøgelser af de tidlige postoperative smerter og fysiske funktionsniveau mellem PA og LA.

LA-proceduren indbefatter som nævnt et direkte kirurgisk traume på hofte-musklerne, nærmere bestemt abduktormusklerne, som skæres af ved deres udspring på lårbensknoglen. Reparation foretages under lukning af operationssåret. Hoftens abduktor-muskler er vigtige for stabiliteten i bækkenet under gang og manglende hofte-abduktor-muskelstyrke er traditionelt forbundet med haltende gang og en positiv Trendelenburgs test( en test der undersøger om patienten kan holde bækkenet vandret, når han/hun står på et ben.) Testen er positiv, hvis bækkenet ”falder” ned til den side hvor benet er løftet)^18, 19^. Maksimal hofte-muskelstyrke (MVC=maximal voluntary contraction) hos THA-patienter er blevet sammenlignet med raske i flere studier og fundet signifikant reduceret blandt THA patienterne^20-22^. Kun to ikke-randomiserede studier har undersøgt forskellene ved hofte-muskelstyrke mellem LA og PA, og resultaterne er ikke entydige ^18, 23^. Et amerikansk studie har imidlertid lavet en sammenlignende gangundersøgelse mellem PA og LA. De fandt at 100% af LA patienterne var karakteriseret ved et unormalt gangmønster, mens 30% af PA patienterne udmærkede sig ved et normalt gangmønster 6 måneder efter operation^24^. De argumenterer for, at det ændrede gangmønster kan være udtryk for en kompensationsmekaniske, der afspejler hofte-abduktor-musklernes svaghed, men de undersøgte det ikke direkte^24^.

Der er i litteraturen flere valide registerundersøgelser^1, 7, 8^, der konkluderer at PA er knyttet til flere proteseledskred og om-operationer pga. dette end LA. Og et studie har vist, at det kan have indflydelse på overlevelsen af ​​protesen^8^. De væsentlige ulemper ved LA, nemlig nedsat funktionsniveau, smerter, lavere patienttilfredshed, ændret gangmønster er i midler tid mindre godt undersøgt. Med denne prospektive randomiserede undersøgelse ønsker vi at undersøge om disse ulemper er mere udtalte hos LA-patienterne og dermed bidrage til bedre viden om de to mest anvendte operative adgang i Danmark. Dette vil bidrage til et mere kvalificeret valg af metode i fremtiden.

# 2.0 Formålet med ph.d.-projektet

Ph.d.-projektet bliver delt op i tre del-studier med følgende formål og hypoteser

## Studie 1- Tidlige resultater af det fysiske funktionsniveau og smerter efter THA operation enten via LA eller PA.

Formålet er at undersøge hypotesen om, at det tidlige fysiske funktionsniveau er dårligere og smerteintensiteten større blandt patienter, der er opereret via LA end patienter opereret via PA

## Studie 2- Effekten af lateral adgang versus bagre adgang på patientrapporterede effektmål op til et år efter THA operation

Formålet er at undersøge hypotesen om, at de patientrapporterede effektmål, inden for det første år efter THA operation, er dårligere blandt patienter opereret via LA end patienter opereret via PA

## Studie 3- Hofte-muskelsvaghed og gangmønstre hos THA-patienter opereret med enten LA eller PA.

Formålet er at undersøge hypotesen om at hofte abduktor-muskelfunktionen er dårligere og gangmønsteret væsentligt ændret fra normal for patienter opereret via LA end patienter opereret via PA, inden for det første år efter THA

3.0 Metoder og materialer
Studie design
Vi vil udføre et prospektivt randomiseret klinisk forsøg med inklusion af 80 patienter med primær hofteslidgigt. Patienterne vil blive randomiseret til primær THA, indsat vha. henholdsvis LA og PA. De kliniske og parakliniske undersøgelser vil foregå under blænding af undersøgerne og patienterne. Foruden patientgruppen, vil vi indsamle ganganalysedatadata fra 20 alders-og køns-matchede personer uden slidgigt, som kontrolgruppe til brug i studie 3.

## Blinding af undersøgelsespersonalet

Undersøgelsespersonalet vil ikke under studieperioden have adgang til patienternes almindelige journal. De vil ikke deltage i operationen eller på nogen måde være involveret i den almindelige pleje af patienterne under indlæggelsen, hvorfor adgang til journalen ikke er nødvendig. Deres rolle vil udelukkenden være i forbindelse med indsamling af projektdata. Patienterne i de to grupper vil ikke adskille sig fra hinanden, fraset selve operationsmetoden, og de vil heller ikke være vidende om hvilken behandling de har fået.

## Randomiseringsproces.

Patienten vil, når der er fundet indikation for operation med THA blive booket til operation ved en sekretær i afdelingen. Hun vil have adgang til de forseglede konvolutter, hvori der ligger et patient ID nummer, samt en tilhørende randomiseringkode. I hht. denne kode vil hun booke patienten til operation ved kirurger, der er specialister i netop enten LA eller PA.

Randomiseringssekvensen er computergenereret og dannet af en tredje person, som ikke ellers har med studiet at gøre. Processen foregår i to trin. Der foretages blokrandomisering i blokke af 20, hvor fordelingen mellem LA og PA bliver balanceret ens. I næste trin foretages en ny randomisering til enten ganganalyser eller ej, inden for hver blok. Dette vil foregå med skæv balance, således at der vil være 2/3 chance for at skulle til ganganalyse og 1/3 chance for ikke at skulle til ganganalyse. Således vil samtlige 40 ganganalysepatienter være fundet inden for de 3 første blokke. Denne skæve balance er udført af hensyn til at vi ønsker tidligt i studiet at foretage en ny sample size beregning på primære outcome GDI i studie 3. Beregningen vil blive foretaget på de første 20 ganganalyser før intervention, hvor GDI og SD beregnes. Såfremt der er behov for yderligere forsøgspersoner for at opnå tilstrækkelig power i studiet, vil vi have mulighed for at inkludere dem ud fra den sidste blok efter samme princip som ovenfor anført.

## Inklusionskriterier

Patienter, med slutstadie af hofte slidgigt i alderen 45-70 år, vil blive inkluderet.

## Eksklusionskriterier

Patienter vil blive ekskluderet hvis de har:

1) slutstadie af slidgigt i flere led(knæ/hofte) end aktuelle hofte, med forventet operation inden for et år.

2) Tidligere operationer på et større led (hofte, knæ) i benene.

3) BMI over 35.

4) Større anatomiske forandringer i hofteleddet, der kræver indsættelse af andre end standard protese komponenter.

5) Neurologiske eller alvorlige medicinske sygdomme, der kompromitterer evnen til at gå 20 meter uden støtte.

6)Svær demens.

7) Manglende evne til at læse eller forstår danske skriftlige og mundtlige vejledninger.

Alle ekskluderede patient vil blive registreret med grunden til udelukkelse.

## Intervention

6 ortopædkirurgiske speciallæger med erfaring i henholdsvis PA eller LA, vil udføre alle operationer. Alle operationer og alle undersøgelser foregår på Odense Universitetshospital. 3 kirurger kommer fra Køge Sygehus, ortopædkirurgisk afdeling H. På Køge Sygehus benyttes udelukkende LA som standart behandling ved THA. De 3 andre kirurger kommer fra Odense Universitets Hospital, hvor alle operationer som standart udføres via PA. Patienterne vil modtage den samme behandling vedrørende patientinformation, operationsfaciliteter, rygmarvsbedøvelse, standardiseret antibiotisk behandling og tromboseprofylakse og smertestillende behandling. Patienterne vil modtage et normalt og standardiseret genoptræningsforløb, udført ved trænede fysioterapeuter. Således vil de to patientgrupper alene være forskellige hvad angår den kirurgiske adgang.

# **4.0 Delstudier**

### Studie 1- Primært effektmål

20 meter gå-test. Patienterne bliver bedt om 4 gange at gå 20 meter mellem to klart synlige linjer markeret på gulvet. Tidsforbrug og antal skridt registreres. Testen er fundet pålidelig og valid for måling af ganghastighed^25^ Ganghastighed har stor betydning for almindelige daglige gøremål, som f.eks. at krydse en trafikeret gade.

Sekundære effektmål

1. "Timed Up and Go"-test (TUG) måler den tid, det tager en person at rejse sig fra en stol gå 3 meter, vende om og gå tilbage til stolen og sætte sig ned igen. TUG er fundet at være en pålidelig og valid test for kvantificering af den funktionelle mobilitet^26, 27^.
2. Repeated chair rise- test (RCR) vurdere styrken af ​​benenes muskler generelt. Testen måler det maksimale antal gange patienten kan rejse sig og sætte sig fra en stol indenfor i 30 sekunder. Testen har vist, at giver en pålidelig og valid måling af den nederste del af kroppens styrke i almindelighed hos aktive ældre voksne (60 +)^28^.
3. Unilateral gentaget knæbøjning tester det maksimale antal knæbøjninger, der kan udføres på 30 sekunder. Testen måler den funktionelle kapacitet over hofte, knæ og ankel. Før testen bestemmes hvor langt patienten skal bøje ned i knæene ved at følge den visuelle linje mellem øjnene og tåspidserne. Den dybeste position, er der, hvor knæene passerer tåspidsrene og dermed bryder den visuelle linje (ca. 30 grader fleksion ). Under testen støttes patienten i at holde balancen af undersøgeren ved at undersøger holder patienten i hænderne. Det raske ben testes først.
4. Behovet for smertestillende medicin vil blive registreret og konverteret til morfin-ækvivalenter. En Visuel Analog Skala for smerter (VAS) vil blive brugt til at vurdere smerter dagligt under indlæggelse ^29^.
5. Efter udskrivelsen vil patienterne udfylde en patient-dagbog, hvor smerterskalaen på HOOS spørgeskemaet(se primært outcome studie 2) bruges, samt i starten VAS-skalaen. Forbruget af smertestillende medicin registreres ligeledes. Registreringer foretages 2,3,4,6,8,10 og 12 uger postoperativt.

## Studie 2- Primært effektmål- Patient rapporterede effektmål (PROM).

Vi vil bruge ”Hip Disability and Osteoarthritis Outcome Score” (HOOS) spørgeskemaet. HOOS omfatter fem subskalaer: 1)Smerter, 2) Andre symptomer, 3) Dagligdags aktiviteter, 4) Sport og rekreative funktioner, og 5) Hofterelateret livskvalitet. HOOS er et pålideligt og valideret instrument til vurdering af THA-patienter^30^. HOOS anbefales til evaluering af patienter med hofte slidgigt som gennemgår kirurgiske indgreb såsom THA ^31^. HOOS er blevet oversat til dansk og valideret^32^.

### Sekundære effektmål

1. EuroQol/EQ-5D er et standardiseret, pålideligt og valideret spørgeskema til at måle livskvalitet og selv-vurderet helbred, og kan anvendes for en bred vifte af sygdomme og behandlinger^33, 34^. EQ-5D anvendes hvor nytte-værdien af en tilstand eller indgreb ønskes vurderet ved at beregne cost-effectiveness eller kvalitetsjusterede leveår, som f.eks. i vurderingen af ​​behandling med THA^35^.
2. University of California Los Angeles aktivitet score (UCLA) er en pointskala med fokus på patientens aktivitetsniveau. Scoren er baseret på en skala fra 1 til 10, der spænder fra helt inaktiv til regelmæssigt at deltage i sport eller fysisk anstrengende arbejde. UCLA bidrager med vigtige kvalitative oplysninger med hensyn til patienternes aktivitet i sammenhæng med andre kliniske målinger^36^.

## Studie 3

### Primært effektmål

En fuld 3D ganganalyse vil blive udført^37^. Ganganalyse er en velbeskreven måde objektivt at måle kompleksiteten af et gangmønster. Det anvendes både i vurdering af THA-patienter og andre patient-populationer^24, 38, 39^. Gang-analysen skaber imidlertid en stor mængde data, som kan være svære at fortolke. Gait Deviation Index (GDI) blev skabt for at præsentere data i én samlet score, for at give en sammenfattende vurdering af patienternes gang sammenlignet med raske personer^40^. GDI beregnes ud fra 15 variabler, der repræsenterer 98% af variationen i gangmønstret. En GDI score på 100 repræsenterer en normal gang. Hvert 10 point under 100 udgør 1 standardafvigelse (SD) fra normal gang^40^. For at vurdere GDI på en THA population, er det nødvendigt at indsamle et reference-materiale på normalt gående kontrolpersoner^40^. I dette studie 20 kontrolpersoner.

### Sekundære effektmål

1. EMG målinger (elektromyografi er en metode til at måle den elektriske aktivitet i musklerne), foretages på hofte musklerne under ganganalysen og muskelstyrkemåling^39, 41^.
2. Maksimal muskelstyrke måles med et dynamometer, med patienten stående^42^. Følgende muskelgrupper undersøges: hofteabduktorerne , hoftefleksermusklerne og hofteekstensermuskelerne.
3. Standardiserede røntgenbilleder af hoften og bækkenet præ-og postoperativt tages rutinemæssigt og vil blive vurderet ud fra bl.a. benlængdeforskel og orienteringen af protesekomponenterne.
4. Hip Range of Motion (ROM) (hoftens passive bevægeudslag) vil blive målt med en gonimeter og noteres i grader.
5. En standardiseret Trendelenburgs test vil blive udført i henhold til Hardcastle et al.^19^.

Der er foretaget styrkeberegning separat på alle delstudier, der viser at vi med de gængse sikkerheds marginer(α=0,05 og β=0,80) kan vise en forskel, såfremt der er en, ved inklusion af 40 patienter i hver gruppe i studie 1 og 2. I studie 3 skal der inkluderes 20 patienter i hver gruppe, se bilag 1.

# 10.0 Reference List

1. **Overgaard S, et al.** Dansk Hoftealloplastik Register- Årsrapport 2009. *Dansk hoftealloplastikregister* 2009.

2. **Learmonth ID, Young C, Rorabeck C** The operation of the century: total hip replacement. *Lancet* 2007;370:1508-19.

3. **Zhang W, Moskowitz RW, Nuki G, Abramson S, Altman RD, Arden N, Bierma-Zeinstra S, Brandt KD, Croft P, Doherty M, Dougados M, Hochberg M, Hunter DJ, Kwoh K, Lohmander LS, Tugwell P** OARSI recommendations for the management of hip and knee osteoarthritis, Part II: OARSI evidence-based, expert consensus guidelines. *Osteoarthritis Cartilage* 2008;16:137-62.

4. **Jolles BM, Bogoch ER** Posterior versus lateral surgical approach for total hip arthroplasty in adults with osteoarthritis. *Cochrane Database Syst Rev* 2004:CD003828.

5. **Palan J, Beard DJ, Murray DW, Andrew JG, Nolan J** Which approach for total hip arthroplasty: anterolateral or posterior? *Clin Orthop Relat Res* 2009;467:473-7.

6. **Edmunds CT, Boscainos PJ** Effect of surgical approach for total hip replacement on hip function using Harris Hip scores and Trendelenburg's test. A retrospective analysis. *Surgeon* 2011;9:124-9.

7. **Havelin LI, Fenstad AM, Salomonsson R, Mehnert F, Furnes O, Overgaard S, Pedersen AB, Herberts P, Karrholm J, Garellick G** The Nordic Arthroplasty Register Association: a unique collaboration between 3 national hip arthroplasty registries with 280,201 THRs. *Acta Orthop* 2009;80:393-401.

8. **Arthursson AJ, Furnes O, Espehaug B, Havelin LI, Soreide JA** Prosthesis survival after total hip arthroplasty--does surgical approach matter? Analysis of 19,304 Charnley and 6,002 Exeter primary total hip arthroplasties reported to the Norwegian Arthroplasty Register. *Acta Orthop* 2007;78:719-29.

9. **Masonis JL, Bourne RB** Surgical approach, abductor function, and total hip arthroplasty dislocation. *Clin Orthop Relat Res* 2002:46-53.

10. **Britton AR, Murray DW, Bulstrode CJ, McPherson K, Denham RA** Pain levels after total hip replacement: their use as endpoints for survival analysis. *J Bone Joint Surg Br* 1997;79:93-8.

11. **Jones CA, Beaupre LA, Johnston DW, Suarez-Almazor ME** Total joint arthroplasties: current concepts of patient outcomes after surgery. *Rheum Dis Clin North Am* 2007;33:71-86.

12. **Nilsdotter AK, Lohmander LS** Patient relevant outcomes after total hip replacement. A comparison between different surgical techniques. *Health Qual Life Outcomes* 2003;1:21.

13. **Ogonda L, Wilson R, Archbold P, Lawlor M, Humphreys P, O'Brien S, Beverland D** A minimal-incision technique in total hip arthroplasty does not improve early postoperative outcomes. A prospective, randomized, controlled trial. *J Bone Joint Surg Am* 2005;87:701-10.

14. **Della Valle CJ, Dittle E, Moric M, Sporer SM, Buvanendran A** A prospective randomized trial of mini-incision posterior and two-incision total hip arthroplasty. *Clin Orthop Relat Res* 2010;468:3348-54.

15. **Maffiuletti NA, Impellizzeri FM, Widler K, Bizzini M, Kain MS, Munzinger U, Leunig M** Spatiotemporal parameters of gait after total hip replacement: anterior versus posterior approach. *Orthop Clin North Am* 2009;40:407-15.

16. **Restrepo C, Parvizi J, Pour AE, Hozack WJ** Prospective randomized study of two surgical approaches for total hip arthroplasty. *J Arthroplasty* 2010;25:671-9.

17. **Nikolajsen L, Brandsborg B, Lucht U, Jensen TS, Kehlet H** Chronic pain following total hip arthroplasty: a nationwide questionnaire study. *Acta Anaesthesiol Scand* 2006;50:495-500.

18. **Downing ND, Clark DI, Hutchinson JW, Colclough K, Howard PW** Hip abductor strength following total hip arthroplasty: a prospective comparison of the posterior and lateral approach in 100 patients. *Acta Orthop Scand* 2001;72:215-20.

19. **Hardcastle P, Nade S** The significance of the Trendelenburg test. *J Bone Joint Surg Br* 1985;67:741-6.

20. **Kiyama T, Naito M, Shinoda T, Maeyama A** Hip abductor strengths after total hip arthroplasty via the lateral and posterolateral approaches. *J Arthroplasty* 2010;25:76-80.

21. **Klausmeier V, Lugade V, Jewett BA, Collis DK, Chou LS** Is there faster recovery with an anterior or anterolateral THA? A pilot study. *Clin Orthop Relat Res* 2010;468:533-41.

22. **Rasch A, Dalen N, Berg HE** Muscle strength, gait, and balance in 20 patients with hip osteoarthritis followed for 2 years after THA. *Acta Orthop* 2010;81:183-8.

23. **Gore DR, Murray MP, Sepic SB, Gardner GM** Anterolateral compared to posterior approach in total hip arthroplasty: differences in component positioning, hip strength, and hip motion. *Clin Orthop Relat Res* 1982:180-7.

24. **Madsen MS, Ritter MA, Morris HH, Meding JB, Berend ME, Faris PM, Vardaxis VG** The effect of total hip arthroplasty surgical approach on gait. *J Orthop Res* 2004;22:44-50.

25. **Nevitt MC., Felson DT, Lester G.** OAI, Protocol for the cohort study. In: 2006.

26. **Podsiadlo D, Richardson S** The timed "Up & Go": a test of basic functional mobility for frail elderly persons. *J Am Geriatr Soc* 1991;39:142-8.

27. **Yeung TS, Wessel J, Stratford PW, MacDermid JC** The timed up and go test for use on an inpatient orthopaedic rehabilitation ward. *J Orthop Sports Phys Ther* 2008;38:410-7.

28. **Jones CJ, Rikli RE, Beam WC** A 30-s chair-stand test as a measure of lower body strength in community-residing older adults. *Res Q Exerc Sport* 1999;70:113-9.

29. **DeLoach LJ, Higgins MS, Caplan AB, Stiff JL** The visual analog scale in the immediate postoperative period: intrasubject variability and correlation with a numeric scale. *Anesth Analg* 1998;86:102-6.

30. **Nilsdotter AK, Lohmander LS, Klassbo M, Roos EM** Hip disability and osteoarthritis outcome score (HOOS)--validity and responsiveness in total hip replacement. *BMC Musculoskelet Disord* 2003;4:10.

31. **Thorborg K, Roos EM, Bartels EM, Petersen J, Holmich P** Validity, reliability and responsiveness of patient-reported outcome questionnaires when assessing hip and groin disability: a systematic review. *Br J Sports Med* 2010;44:1186-96.

32. **Beyer N, Thorborg K, Vinther A Translation and Cross-Cultural Adaptation of the Danish Version of the Hip Dysfunction and**

**Osteoarthritis Outcome Score 2.0 (HOOS 2.0)**. In: 2008.

33. EuroQol--a new facility for the measurement of health-related quality of life. The EuroQol Group. *Health Policy* 1990;16:199-208.

34. **Brooks R** EuroQol: the current state of play. *Health Policy* 1996;37:53-72.

35. **Ostendorf M, van Stel HF, Buskens E, Schrijvers AJ, Marting LN, Verbout AJ, Dhert WJ** Patient-reported outcome in total hip replacement. A comparison of five instruments of health status. *J Bone Joint Surg Br* 2004;86:801-8.

36. **Beaule PE, Dorey FJ, Hoke R, Le DM, Amstutz HC** The value of patient activity level in the outcome of total hip arthroplasty. *J Arthroplasty* 2006;21:547-52.

37. **Kadaba MP, Ramakrishnan HK, Wootten ME** Measurement of lower extremity kinematics during level walking.

38. **Foucher KC, Hurwitz DE, Wimmer MA** Preoperative gait adaptations persist one year after surgery in clinically well-functioning total hip replacement patients. *J Biomech* 2007;40:3432-7.

39. **Pospischill M, Kranzl A, Attwenger B, Knahr K** Minimally invasive compared with traditional transgluteal approach for total hip arthroplasty: a comparative gait analysis. *J Bone Joint Surg Am* 2010;92:328-37.

40. **Schwartz MH, Rozumalski A** The Gait Deviation Index: a new comprehensive index of gait pathology. *Gait Posture* 2008;28:351-7.

41. **Perron M, Malouin F, Moffet H, McFadyen BJ** Three-dimensional gait analysis in women with a total hip arthroplasty. *Clin Biomech (Bristol , Avon )* 2000;15:504-15.

42. **Jensen C, Overgaard S, Aagaard P** Assessment of Maximal Hip and Knee Mechanical Muscle Impairment in THA patients: Reliability and Agreement.
