# Supplementary figures and images for: Radiographic cup position following posterior and lateral approach to total hip arthroplasty. An explorative randomized controlled trial
Source: PLoS One. 2018 Jan 29;13(1):e0191401. doi: 10.1371/journal.pone.0191401 (PMC5788339; doi:10.1371/journal.pone.0191401)

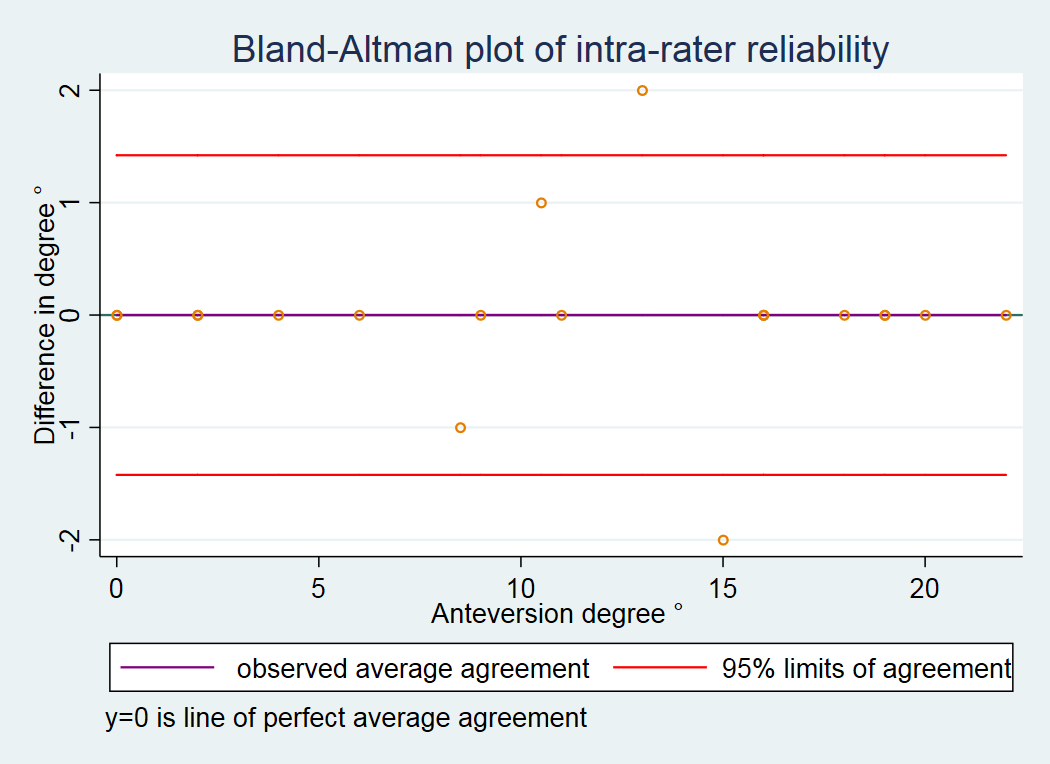

Supplement: S1 Fig — (TIF) [file pone.0191401.s008.tif]

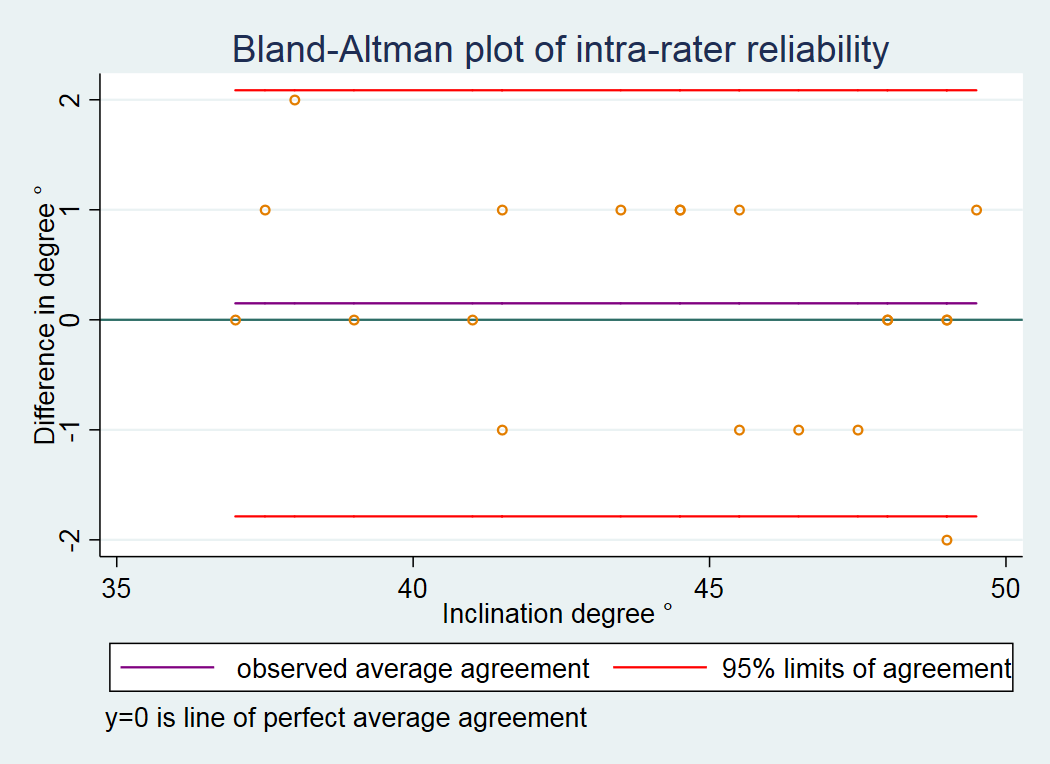

Supplement: S2 Fig — (TIF) [file pone.0191401.s009.tif]
